# Perioperative outcomes and continence following robotic-assisted radical cystectomy with mainz pouch II urinary diversion in patients with bladder cancer

**DOI:** 10.1186/s12885-024-11874-x

**Published:** 2024-01-24

**Authors:** Suoshi Jing, Enguang Yang, Zuoxi Luo, Yunxin Zhang, Hui Ding, Li Yang, Zhilong Dong, Panfeng Shang, Zhongjin Yue, Gongjin Wu, Junsheng Bao, Junqiang Tian, Jiaji Wang, Nan Xiao, Zhiping Wang

**Affiliations:** 1 Institute of Urology, Key Laboratory of Gansu Province for Urological Diseases, Lanzhou University Second Hospital, Gansu Nephro-Urological Clinical Center, 730030 Lanzhou, China; 2 Department of Urology, The First Hospital of Lanzhou University, 730030 Lanzhou, China

**Keywords:** Robotic-assisted radical cystectomy, Mainz pouch II, Urinary diversion, Continence, Bladder cancer

## Abstract

**Purpose:**

To present the widely unknown perioperative outcomes and continence status of bladder cancer patients following robotic-assisted radical cystectomy (RARC) with Mainz pouch II urinary diversion (UD).

**Materials and methods:**

From November 2020 to December 2023, 37 bladder cancer patients who underwent RARC with Mainz pouch II UD were retrospectively assessed (ChiCTR2300070279). The results, which included patient demographics, perioperative data, continence, and complications (early ≤ 30 days and late ≤ 30 days) were reported using the RC-pentafecta criteria. RC-pentafecta criteria included ≥ 16 lymph nodes removed, negative soft tissue surgical margins, absence of major (Grade III–IV) complication at 90 days, absence of clinical recurrence at ≤ 12 months, and absence of long-term UD-related sequelae. A numeric rating scale assessed patient satisfaction with urinary continence 30 days after surgery. The validated Patient Assessment of Constipation Symptoms (PAC-SYM) questionnaire was used to evaluate bowel function. The Kaplan-Meier curve was used to evaluate overall survival (OS).

**Results:**

Of the 37 patients evaluated over a median (range) follow-up period of 23.0 (12.0-36.5) months. The median (range) age was 65 (40–81) years. The median (range) time to urinary continence after surgery was 2.3 (1.5-6) months. Of the 37 patients, 31 (83.8%) were continent both during the day and at night, 34 (91.9%) were continent during the day, 32 (86.5%) were continent at night, 35 (94.6%) were satisfied with their urinary continence status, and 21 (56.8%) were very satisfied. The mean (range) voiding frequency was 6 (4–10) during the day and 3 (2-5.5) at night. The mean (range) PAC-SYM total score was 9.50 (4.00–15.00). In 12 (32.4%) of the patients, RC-pentafecta was achieved, and achieving RC-pentafecta was linked to better satisfaction scores (7.3 vs. 5.5, *p* = 0.034). There was no significant difference between RC-pentafecta and No RC-pentafecta groups in terms of OS (25.6 vs. 21.5 months, *p* = 0.16). 7 (19.4%) patients experienced late complications.

**Conclusions:**

Mainz pouch II UD following RARC in bladder cancer patients results in a satisfactory continence rate. Achieving RC-pentafecta was correlated with better satisfaction scores. The intracorporeal approach to Mainz pouch II UD is beneficial for female patients due to its reduced invasiveness.

**Trial registration:**

ChiCTR2300070279; Registration: 07/04/2023, Last updated version: 01/06/2023. Retrospectively registered.

## Background

Muscle-invasive bladder cancer (MIBC) has a poor prognosis, with a 5-year cancer-specific mortality rate of 86%. Depending on cisplatin eligibility, the first-line treatment for patients with nonmetastatic MIBC and very high-risk nonmuscle-invasive (NMIBC) as well as for selected patients with NMIBC, such as patients with NMIBC progressing to MIBC and patients with bacillus Calmette-Guérin (BCG) unresponsive or BCG-relapsing high-grade tumors [1] is radical cystectomy (RC) with pelvic lymph node dissection (PLND) and urinary diversion (UD) [2]. Furthermore, less than 30% of patients who received trimodality therapy (TMT) using maximal transurethral resection of bladder tumor (TURBt) and chemoradiation therapy [3] will still require RC due to tumor progression [4]. Robotic-assisted RC (RARC) has been increasingly used because of its minimal invasiveness [5], and incontinent UD is used for therapeutic purposes whereas continent UD is used for palliative purposes. Orthotopic neobladder (ONB) is the main type of continent UD [3, 6, 7]; the procedure is complicated and limited by natural contraindications such as urethral invasion. However, Mainz pouch II UD (sigma rectum pouch), another type of procedure used to restore continence, is a relatively simple and quick procedure with considerable advantages for certain patients [8, 9]. In our clinical practice, the Mainz pouch II UD is thus the principal UD type, and it is employed in surgeries ranging from open to total intracorporeal [10, 11]. There is a paucity in urologic literature regarding outcomes of Mainz pouch II UD especially following RARC. To examine perioperative outcomes and continence status following RARC, we perform RARC with Mainz pouch II UD in patients with MIBC.

## Materials and methods

### Study subjects

With the approval of the medical ethics committee of Lanzhou University Second Hospital, from November 2020 to December 2023, 37 patients with bladder cancer undergoing RARC and Mainz pouch II UD were prospectively analyzed. The study was registered in the Chinese Clinical Trial Registry (ChiCTR2300070279). Indications for RARC have been previously described [10, 11]. Patient demographics, perioperative data, complications (early ≤ 30 days and late>30 days) classified according to Clavien-Dindo, and continence rate were recorded. Patient demographics data comprised sex, age, American Society of Anesthesiologists (ASA) score, body mass index (BMI), comorbidity (defined as coronary atherosclerotic heart disease, hypertension, or diabetes), age-adjusted Charlson comorbidity index (ACCI) [12, 13], and smoking status. Perioperative data included operative time, estimated blood loss (EBL), intraoperative transfusion rate, tumor size (the maximum diameter of tumor), total LN yield removed, postoperative length of hospital stay (LOS), and pathologic parameters.

### Patient satisfaction with urinary continence

Continence was defined as pad-free or need of up to one safety pad [14–16] and continence rate was recorded for daytime and nighttime. Patient satisfaction with urinary continence was measured 30 days following surgery using an unvalidated numeric rating scale ranging from 0 to 10 points. Patients were asked to rate themselves based on their urinary continence one month following surgery. The higher the score, the greater the patient’s satisfaction with their urinary continence. The following is how the satisfaction scores are interpreted: 0 points = dissatisfied, 1–3 points = moderately satisfied, 4–6 points = satisfied, and 7–10 points = very satisfied.

#### Preoperative bowel preparation

The day before surgery, patients were given 2–4 bags of polyethylene glycol electrolyte powder dissolved in 1000–2000 mL warm water, and asked to drink 600 mL at first, followed by 250 mL every 10–15 min, and they had to fast after 8 p.m. On the morning of the operation, patients underwent a cleansing enema once.

#### Thromboembolism prophylaxis policy

To prevent venous thromboembolism, patients were given intermittent pneumatic leg compression once a day for 10–15 min before getting out of bed.

### Surgical technique

#### ***Patient position***

After general anesthesia, the patient was placed in the Trendelenburg position (25–30°), and bilateral leg bandages were applied. After disinfection of the surgical area, catheterization was performed.

#### ***Port placement***

RARC was performed with the da Vinci Xi system (Intuitive Surgical, Inc., Sunnyvale, CA, USA). The 8-mm camera port was placed 5 cm above the umbilicus and secured to prevent air leakage. Then, the other ports were placed under direct vision of the camera; the pneumoperitoneum was 14–15 mm Hg. Two 8-mm robotic ports on either side of the camera port were placed two cm below the camera port level and 8 cm from the midline, and a third 8-mm robotic port on the right side was placed level with the umbilicus 16 cm from the midline. An assistant port on the left side of the camera port was placed three cm above the anterior superior iliac spine through a 12-mm Trocar.

#### Standard RARC

##### Identification and dissection of the ureters

After being identified at the crossing of the common iliac artery, the ureters were dissected to the bladder and cut between two Hem-o-Lok clips. During this procedure, adequate periureteric tissue was maintained. The distal ureteric margins were sent for pathological examination.

##### RC after PLND

The anatomic landmarks of bladder posterior dissection include the vas deferens, seminal vesicles, and Denonvilliers fascia. Opening the peritoneum at the rectovesical pouch exposed the seminal vesicles and vas deferens, and then the Denonvilliers fascia was exposed behind the seminal vesicles. Retroprostatic dissection was performed at the surgical plane between the Denonvilliers fascia and the rectum. Subsequently, the Retzius space was developed by incising the peritoneum lateral to the medial umbilical ligaments and the endopelvic fascia was exposed by sharp and blunt dissection. The endopelvic fascia was then incised, and the lateral surface of the prostate was separated from the levator ani muscle, the puboprostatic ligament, the lateral pedicles of the bladder and the prostate, and the venous plexus was ligated and dissected using Hem-o-Lok clips. The urachus and the median umbilical ligaments were cut to expose the anterior bladder space. The dorsal vein complex was isolated and sutured using 2 − 0 sutures, and the urethra was then transected at the prostatic apex. The specimen was separated immediately and placed in a large organ bag to be removed later at the end of the procedure via the vagina or abdominal incision.

##### Intracorporeal mainz pouch II UD

A length of 12 cm sigma and rectum were selected with the rectosigmoid junction as the midpoint and reconstructed in an inverted manner. The wall of the bowel was opened by four cm at the bottom of the U-shape. The Mainz pouch II was created by the side-to-side anastomosis of the selected sigma and rectum using two 60-mm Endo-GIA staplers through the 12-mm assistant trocar. One arm of the Endo-GIA staplers was inserted into the sigma, and the other arm was placed in the rectum. The side-to-side anastomosis was completed at the time of closing the two arms, and then, the anterior and posterior walls of the Mainz pouch II were created as a 12 cm rectosigmoid reservoir as created. After the left ureter was tunneled to the right side under the sigmoid mesentery, the right and left ureter were pulled into the peritoneal cavity through a right and left paracolonic peritoneal incision, respectively, with no kink or tension. After the ureteric margin was cut and sent for pathological examination, the ureteric stump was cut lengthwise one cm and evaginated to form a papilla as an antireflux mechanism. Two punctures were made in the posterior wall of the Mainz pouch II, and the ureters were pulled into the pouch through the punctures and fixed using 4/0 absorbable sutures via the mucosa-to-mucosa technique. Two 6 F Mono-J stents were inserted into the bilateral ureters, and the other ends were led out with the rectal tube and fixed to the skin. The pouch was finally closed using a 4/0 absorbable suture.

##### Open mainz pouch II UD

The procedure was converted to open after the specimen was removed, and the surgical procedure of Mainz pouch II UD was performed as described previously [10, 11].

### Definition of RC-pentafecta

RC-pentafecta patients were defined as those who concurrently achieved ≥ 16 LNs removed, negative soft tissue surgical margins (STSMs), absence of major (Grade III–IV) complication at 90 days, absence of clinical recurrence at ≤ 12 months, and absence of long-term UD-related sequelae [17, 18].

### Patient assessment of constipation symptoms (PAC-SYM) questionnaire

The validated PAC-SYM questionnaire was used to evaluate bowel function. The 12-item PAC-SYM questionnaire has a 5-point Likert Scale scoring system (0–4: absent - very severe). Three symptom subscales can be used to group questions 1–4, 5–7, and 8–12: abdominal, rectal, and stool, respectively. There is no stated cut-off score, and a total score can vary from 0 to 48. Poorer bowel function is correlated with higher PAC-SYM scores [19].

### Statistical analysis

Values for continuous variables are given as the mean standard deviation (SD) and median (range), while categorical variables are given as frequencies and percentages. Using R 4.3.0, statistical analysis was carried out. For statistical description and difference analysis, the “gtsummary” package was utilized. The Kaplan-Meier log-rank test curve analysis was carried out using the “survival” and “survminer” packages. A statistically significant result for a two-tailed test was *P* < 0.05.

## Results

### Patient demographics

Among the 37 included patients, 12 (32.4%) attained the RC-pentafecta (RC-pentafecta group), while 25 (67.6%) did not (No RC-pentafecta group). All patients underwent RARC, including 2 (5.4%) patients who underwent intracorporeal UD and 35 (94.6%) patients who underwent open Mainz pouch II UD, and 10.8% received neoadjuvant therapy. The median (range) age was 65 (40–81) years, and the BMI was 24 (18–29) kg/m². Of the 37 patients, 29 (78.4%) were male, and 12 (32.4%) had a smoking history. In all, 33 (89.2%) and 4 (10.8%) patients had ASA scores of II and III, respectively, and 24 patients (64.9%), 12 patients (32.4%) and 1 patient (2.7%) had ACCI scores of < or = 2, 3–5, and > 5, respectively. A total of 12 (32.4%) patients underwent TURBt, and all of them had experienced tumor relapse, as outlined in Table 1.

**Table 1:** The patients’ baseline characteristics

Characteristic		Overall	RC-pentafecta	No RC-pentafecta	*p*-value
Patients	*n* (%)	37(100)	12(32.4)	25(67.6)	
Age, years	mean (SD)	64.7 (9.78)	61.4 (8.86)	66.3 (9.98)	0.158^a^
	median (range)	65.00 (40.00–81.00)	61.50 (49.00–76.00)	66.00(40.00–81.00)	
BMI, kg/m^2^	mean (SD)	23.86 (2.69)	24.50 (2.71)	23.56 (2.68)	0.31^a^
	median (range)	24.00 (18.00–29.00)	24.50 (19.00–29.00)	24.00 (18.00- 26.28)	
Gender	*n * (%)				0.394^b^
Male		29.00 (78.38)	8.00 (66.67)	21.00 (84.00)	
Female		8.00 (21.62)	4.00 (33.33)	4.00 (16.00)	
ASA score	*n * (%)				0.282^b^
ASA II		33.00 (89.19)	12.00 (100.00)	21.00 (84.00)	
ASA III		4.00 (10.81)	0.00 (0.00)	4.00 (16.00)	
History of smoking	*n * (%)				0.468^b^
No		25.00 (67.57)	7.00 (58.33)	18.00 (72.00)	
Yes		12.00 (32.43)	5.00 (41.67)	7.00 (28.00)	
Neoadjuvant therapy	*n * (%)				0.282^b^
No		33.00 (89.19)	12.00 (100.00)	21.00 (84.00)	
Yes		4.00 (10.81)	0.00 (0.00)	4.00 (16.00)	
Comorbidity*	*n * (%)				0.315^b^
No		22.00 (59.46)	6.00 (50.00)	16.00 (64.00)	
One kind		12.00 (32.43)	5.00 (41.67)	7.00 (28.00)	
Two kinds		2.00 (5.41)	0.00 (0.00)	2.00 (8.00)	
Three kinds		1.00 (2.70)	1.00 (8.33)	0.00 (0.00)	
ACCI score	*n * (%)				0.803^b^
< or = 2		24.00 (64.86)	9.00 (75.00)	15.00 (60.00)	
3–5		12.00 (32.43)	3.00 (25.00)	9.00 (36.00)	
>or = 5		1.00 (2.70)	0.00 (0.00)	1.00 (4.00)	
Prior TURBt					0.711^b^
No		25.00 (67.57)	9.00 (75.00)	16.00 (64.00)	
Yes		12.00 (32.43)	3.00 (25.00)	9.00 (36.00)	
No. Prior TURBt	*n * (%)				0.651^b^
0		25.00 (67.57)	9.00 (75.00)	16.00 (64.00)	
1		6.00 (16.22)	1.00 (8.33)	5.00 (20.00)	
2		4.00 (10.81)	1.00 (8.33)	3.00 (12.00)	
3		1.00 (2.70)	1.00 (8.33)	0.00 (0.00)	
4		1.00 (2.70)	0.00 (0.00)	1.00 (4.00)	
Relapse after TURBt	*n * (%)				> 0.999^b^
No		25.00 (67.57)	8.00 (66.67)	17.00 (68.00)	
Yes		12.00 (32.43)	4.00 (33.33)	8.00 (32.00)	

ASA, American Society of Anesthesiologists. ACCI, age-adjusted Charlson comorbidity index. BMI, body mass index. SD, standard deviation. TURBt, transurethral resection of bladder tumor. *Comorbidity refers to coronary atherosclerotic heart disease, hypertension, or diabetes. a, Wilcoxon rank sum test. b, Fisher’s exact test.

### Perioperative outcomes

**Fig. 1:**
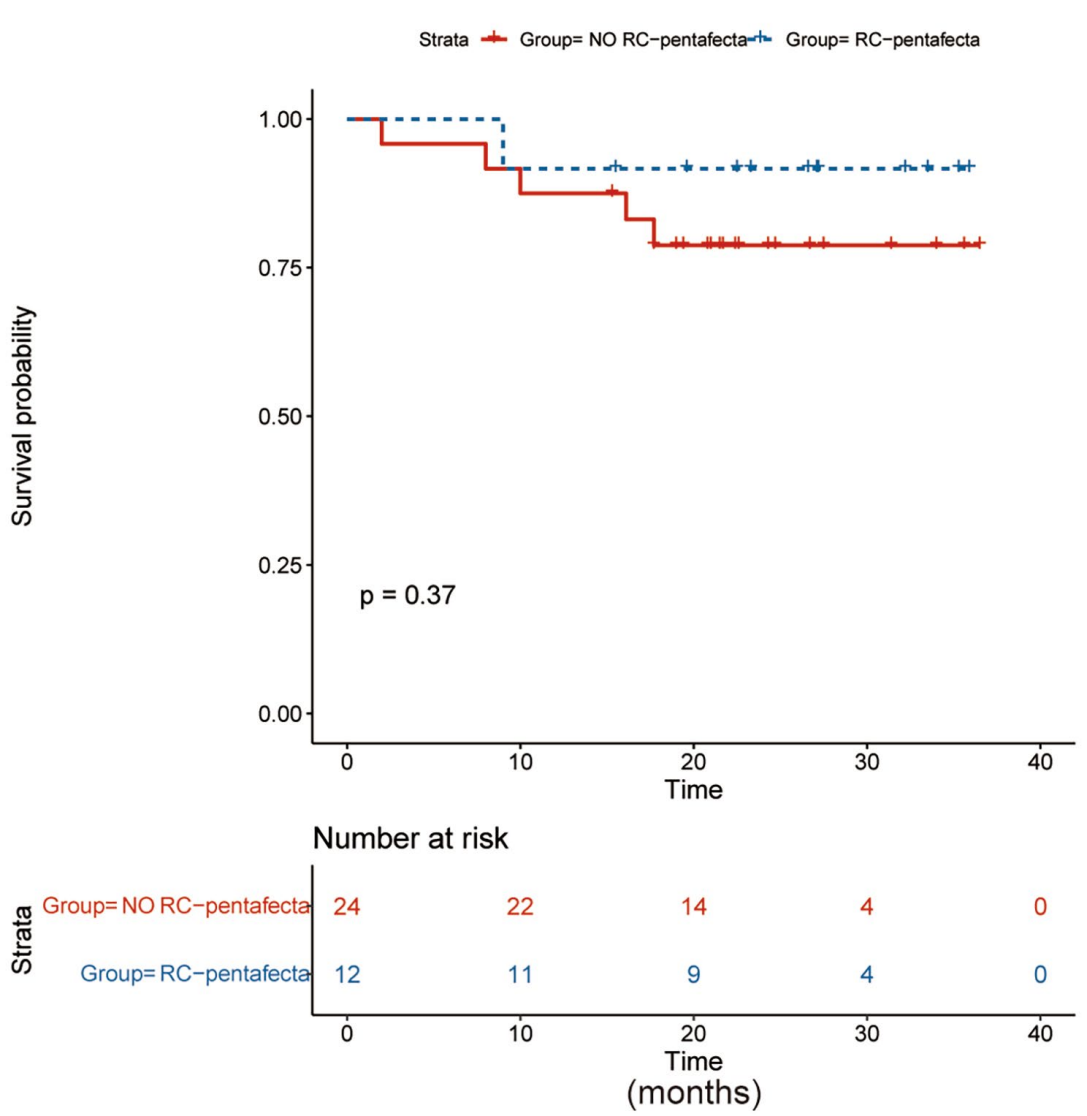
Kaplan–Meier survival curves comparing over survival between the RC- pentafecta and No RC-pentafecta groups

With a median (range) follow-up period of 23.0 (12.00-36.5) months, one patient was lost at a subsequent follow-up, and overall mortality was observed in 6 (16.7%) patients, 4 of whom died from cancer. Overall survival (OS) did not differ statistically between the RC-pentafecta and No RC-pentafecta groups (25.6 vs. 21.5 months, *p* = 0.16), as shown in Fig. 1. The mean (range) postoperative LOS was 13 (7–42) days, tumor size was 3.0 (2.0–9.0) cm, operative time was 475 (300–750) min and EBL was 300 (100–2000) mL. Postoperative complications occurred in 10 (27.8%) patients, including early (≤ 30 days) complications in 3 (8.3%) patients and late (> 30 days) complications in 7 (19.4%) patients, and 8 (22.9%) Clavien‒Dindo grade III-V complications, as listed in Table 2. Two days after surgery, one patient experienced an acute pulmonary embolism, was transferred to the Intensive Care Unit, treated for three days, and finally recovered. Eight days after surgery, one patient developed an incision infection and was treated conservatively. Twenty days after surgery, one patient had prolonged ileus and underwent surgical intervention.

**Table 2:** Perioperative outcomes

Variable		Overall	RC-pentafecta	No RC-pentafecta	*p*-value	
Patients	*n* (%)	37(100)	12(32.4)	25(67.6)		
Follow-up, months	mean (SD)	24.2(6.90)	25.9(7.68)	23.4 (6.48)	0.303^a^	
	median (range)	23.0 (12.00-36.5)	26.8 (12.00-35.90)	22.1 (12.00-36.50)		
Type of UD	*n* (%)				> 0.999^b^	
Open UD		35.00 (94.59)	12.00 (100.00)	23.00 (92.00)		
Intracorporeal UD		2.00 (5.41)	0.00 (0.00)	2.00 (8.00)		
Time to urinary continence, months	mean (SD)	2.74 (1.41)	2.45 (1.17)	2.87 (1.52)	0.511^a^	
	median (range)	2.3 (1.50-6.00)	2.50 (1.50–5.50)	2.00 (1.50-6.00)		
Operative time, min	mean (SD)	479.16 (112.08)	507.50 (125.00)	465.56 (105.29)	0.407^a^	
	median (range)	475.00 (300.00-750.00)	467.50 (360.00-750.00)	475.00 (3300.00-660.00)		
EBLs, mL	mean (SD)	452.97 (388.36)	421.67 (292.01)	468.00 (431.78)	0.882^a^	
	median (range)	300.00 (100.00–2,000.00)	350.00 (100.00–1,000.00)	300.00 (100.00–2,000.00)		
Tumor size, cm	mean (SD)	3.56 (1.77)	3.19 (1.02)	3.73 (2.03)	0.793^a^	
	median (range)	3.00 (2.00–9.00)	3.00 (2.00–5.00)	3.00 (2.00–9.00)		
Intraoperative transfusion	*n* (%)				> 0.999^b^	
No		29.00 (78.38)	10.00 (83.33)	19.00 (76.00)		
Yes		8.00 (21.62)	2.00 (16.67)	6.00 (24.00)		
No. of LNs removed	mean (SD)	14.84 (9.43)	18.50 (2.65)	13.08 (10.97)	0.102^a^	
	median (range)	11.00 (4.00-4100)	17.50 (16.00–23.00)	9.00 (4.00–41.00)		
Voiding frequency by day	mean (SD)	6.17 (1.41)	6.36 (1.73)	6.08 (1.27)	0.745^a^	
	median (range)	6.00 (4.00–10.00)	6.00 (4.00–8.00)	6.00 (4.00–10.00)		
Voiding frequency at night	mean (SD)	3.40 (0.89)	3.14 (0.50)	3.52 (1.01)	0.279^a^	
	median (range)	3.00 (2.00-5.50)	3.00 (2.50-4.00)	3.50 (2.00-5.50)		
Urinary continence both day and night	*n* (%)				0.641^b^	
No		6.00 (16.22)	1.00 (8.33)	5.00 (20.00)		
Yes		31.00 (83.78)	11.00 (91.67)	20.00 (80.00)		
Urinary ontinence by day	*n* (%)				> 0.999^b^	
No		3.00 (8.11)	1.00 (8.33)	2.00 (8.00)		
Yes		34.00 (91.89)	11.00 (91.67)	23.00 (92.00)		
Urinary continence at night	*n* (%)				> 0.999^b^	
No		5.00 (13.51)	1.00 (8.33)	4.00 (16.00)		
Yes		32.00 (86.49)	11.00 (91.67)	21.00 (84.00)		
Satisfaction scores	mean (SD)	6.11 (2.46)	7.33 (1.61)	5.52 (2.60)	0.034^a^	
	median (range)	7.00 (0.00–9.00)	8.00 (3.00–9.00)	6.00 (0.00–9.00)		
Satisfaction	*n* (%)				0.262^b^	
Dissatisfied (0 points)		2.00 (5.41)	0.00 (0.00)	2.00 (8.00)		
Moderately satisfied (1–3 points)		5.00 (13.51)	0.00 (0.00)	5.00 (20.00)		
Satisfied (4–6 points)		9.00 (24.32)	3.00 (25.00)	6.00 (24.00)		
Very satisfied (7–10 points)		21.00 (56.76)	9.00 (75.00)	12.00 (48.00)		
Postoperative LOS, days	mean (SD)	15.11 (8.50)	15.75 (8.24)	14.80 (8.78)	0.589^a^	
	median (range)	13.00 (7.00–42.00)	14.00 (8.00–35.00)	13.00 (7.00–42.00)		
Complications	*n* (%)				0.438^b^	
No		26.00 (72.22)	10.00 (83.33)	16.00 (66.67)		
Yes		10.00 (27.78)	2.00 (16.67)	8.00 (33.33)		
Early complications	*n* (%)				> 0.999^b^	
No		33.00 (91.67)	11.00 (91.67)	22.00 (91.67)		
Yes		3.00 (8.33)	1.00 (8.33)	2.00 (8.33)		
Late complications	*n* (%)				0.384^b^	
No		29.00 (80.56)	11.00 (91.67)	18.00 (75.00)		
Yes		7.00 (19.44)	1.00 (8.33)	6.00 (25.00)		
Clavien-Dindo	*n* (%)				0.378^b^	
Grade I-II		2.00 (20.00)	1.00 (50.00)	1.00 (12.50)		
Grade III-V		8.00 (80.00)	1.00 (50.00)	7.00 (87.50)		
UD-related sequelae free	*n* (%)				0.646^b^	
Yes		31.00 (86.11)	11.00 (91.67)	20.00 (83.33)		
No		5.00 (13.89)	1.00 (8.33)	4.00 (16.67)		
Major complications free within 90 days	*n* (%)				0.079^b^	
Yes		30.00 (83.33)	12.00 (100.00)	18.00 (75.00)		
No		6.00 (16.67)	0.00 (0.00)	6.00 (25.00)		
PAC-SYM total score	mean (SD)	9.73 (2.95)	8.55 (2.46)	10.42 (3.04)	0.093^a^	
	median (range)	9.50 (4.00–15.00)	9.00 (4.00–12.00)	11.00 (4.00–15.00)		
Abdominal subscore	mean (SD)	3.10(1.35)	2.73 (1.01)	3.32 (1.49)	0.256^a^	
	median (range)	3.00 (1.00–6.00)	3.00 (1.00–4.00)	3.00 (1.00–6.00)		
Rectal subscore	mean (SD)	2.9 (1.30)	2.45(1.44)	3.16 (1.17)	0.703^a^	
	median (range)	2.50 (1.00–6.00)	2.00 (1.00–6.00)	3.00 (2.00–6.00)		
Stool subscore	mean (SD)	3.60 (1.55)	3.18 (1.17)	3.84 (1.71)	0.267^a^	
	median (range)	3.00 (1.00–7.00)	3.00 (2.00–6.00)	4.00 (1.00–7.00)		
Overall survival, months	mean (SD)	22.85 (8.37)	25.64 (8.20)	21.45 (8.26)	0.16^a^	
	median (range)	22.45 (2.00-36.50)	26.85 (9.00-35.90)	21.25 (2.00-36.50)		
Overall mortality	*n* (%)	6.00 (16.67)	1.00 (8.33)	5.00 (20.83)	> 0.999^a^	

EBL, estimated blood loss. LOS, length of hospital stay. LN, lymph node. PAC-SYM. Patient Assessment of Constipation Symptoms. SD, standard deviation. UD, urinary diversion. a, Wilcoxon rank sum test. b, Fisher’s exact test.

In terms of late complications, three patients developed entero-cutaneous fistulas, two of whom underwent enterostomy and one of whom received conservative treatment (percutaneous drainage), all of the patients are now well; one patient experienced ureterohydronephrosis, which was treated with a Holmium: YAG laser endoureterotomy and balloon dilation, and the nephrostomy tube was removed after four weeks; one patient experienced septic shock and was treated conservatively; one patient had bilateral ureteroenteric strictures, which were treated with bilateral 8 F stents implanted following balloon dilation; and one patient developed urosepsis and was admitted to the Intensive Care Unit for three days before recovering.

### Continence

The median (range) time to urinary continence after surgery was 2.3 (1.5-6) months. 31 (83.8%) patients were continent both during the day and at night, 34 (91.9%) were continent during the day and 32 (86.5%) at night, 35 (94.5%) patients were satisfied with their urinary continence status. Satisfaction scores were found to be higher in the RC-pentafecta group (7.3 vs. 5.5, *p* = 0.034). The median (range) voiding frequency was 6 (4–10) during the day and 3 (2-5.5) at night. In all, 2 (5.4%), 5 (13.5%), 9 (24.3%), and 21 (56.8%) patients had satisfaction scores of 0 points (dissatisfied), 1–3 points (moderately satisfied), 4–6 points (satisfied) and 7–10 points (very satisfied), respectively, as summarized in Table 2.

### Bowel function outcomes

The median(range) bowel function scores for the PAC-SYM total score, abdominal, rectal, and stool subscores were 9.50 (4.00–15.00), 3.00 (1.00–6.00), 2.50 (1.00–6.00), and 3.00 (1.00–7.00), respectively. In terms of the PAC-SYM total score (8.55 vs. 10.42 months, *p* = 0.093), no significant difference was seen between the RC-pentafecta and No RC-pentafecta groups, as indicated by Table 2.

### Oncological outcomes

There were no positive margins related to the procedure. The mean (range) total LN yield was 11 (4–41), and 6 (16.2%) patients had positive LN. Urothelial carcinoma was observed in 36 (97.3%) patients and squamous cell carcinoma in one (2.7%), as detailed in Table 3.

**Table 3:** Oncological outcomes

Variable		Overall	RC-pentafecta	No RC-pentafecta	*p*-value
Patients	*n* (%)	37.00 (100.00)	12.00 (100.00)	25.00 (100.00)	
STSMs	*n* (%)	37.00 (100.00)	12.00 (100.00)	25.00 (100.00)	
Positive LNs	*n* (%)	6.00 (16.22)	1.00 (8.33)	5.00 (20.00)	0.641^a^
No. positive LNs	mean (SD)	0.32 (0.85)	0.33 (1.15)	0.32 (0.69)	0.463^b^
	median (range)	0.00 (0.00–4.00)	0.00 (0.00–3.00)	0.00 (0.00–4.00)	
Clinical recurrence free within 1 year	*n* (%)				0.646^a^
No		31.00 (86.11)	11.00 (91.67)	20.00 (83.33)	
Yes		5.00 (13.89)	1.00 (8.33)	4.00 (16.67)	
Pathological_type	*n* (%)				> 0.999^a^
Urothelial carcinoma		36.00 (97.30)	12.00 (100.00)	24.00 (96.00)	
Squamous cell carcinoma		1.00 (2.70)	0.00 (0.00)	1.00 (4.00)	
Pathological T-stage	*n* (%)				0.443^a^
pT1		11(29.7)	3.00 (25.00)	8.00 (32.00)	
pT2		16(43.2)	6.00 (50.00)	10.00 (40.00)	
pT3		6(16.2)	3.00 (25.00)	3.00 (12.00)	
pT4		4(10.8)	0.00 (0.00)	4.00 (16.00)	
pN stage	*n* (%)				0.808^a^
pN0		31(83.8)	11.00 (91.67)	20.00 (80.00)	
pN1		2(5.4)	0.00 (0.00)	2.00 (8.00)	
pN2		4(10.8)	1.00 (8.33)	3.00 (12.00)	
pM stage	*n* (%)				
pM0		0(0)	0(0)	0(0)	
Pathological grading	*n* (%)				0.659^a^
Low grade		7(18.9)	3.00 (25.00)	4.00 (16.00)	
High grade		30(81.1)	9.00 (75.00)	21.00 (84.00)	
Angiolymphatic invasion	*n* (%)				0.149^a^
Negative		24.00 (64.86)	10.00 (83.33)	14.00 (56.00)	
Positive		13.00 (35.14)	2.00 (16.67)	11.00 (44.00)	
Recurrence rate	*n* (%)				0.224^a^
No		28.00 (77.78)	11.00 (91.67)	17.00 (70.83)	
Yes		8.00 (22.22)	1.00 (8.33)	7.00 (29.17)	
Secondary neoplasm	*n* (%)	0(0)	0(0)	0(0)	

LN, lymph node. STSMs, negative soft tissue surgical margins. SD, standard deviation. a, Fisher’s exact test. b, Wilcoxon rank sum test.

## Discussion

According to our findings, RARC with Mainz pouch II UD was related to a high continence rate in individuals with MIBC, and attaining RC-pentafecta is related to higher satisfaction scores. Additionally, our research revealed that the intracorporeal approach to Mainz pouch II UD after RARC, which involves removing the specimen via the vagina, is a better option for female patients. This approach eliminates the need for a major abdominal incision and is less invasive.

The Mainz pouch II UD is a good alternative to other types of continent UD that provides desirable continence and quality of life as well as good results in terms of mortality and morbidity [9, 20].

Mainz pouch II is a low-pressure reservoir, and the mean sigmoid pressure decreases from 20 cm H_2_O preoperatively to 6 and 6.5 cm H_2_O at 3 and 6 months postoperatively, respectively. The mean pressure is 8.7 cm H_2_O, the average capacity is approximately 500 mL, and the pressure and capacity of the neobladder are similar to those of a normal bladder. Mainz pouch II UD preserves continence by the anal sphincter, and the function of the anal sphincter should therefore be evaluated preoperatively [7]. Rectodynamic analysis, urodynamics, Medtronic rectal manometry, or retention enema can be used to evaluate anal sphincter function [10, 21]. In our study, the anal sphincter function test was administered to all patients using a 500 mL saline retention enema in an upright position for an hour or 350 mL for three hours. Mainz pouch II UD was considered inappropriate if one was unable to persevere. Additionally, a colonoscopy was also performed on each patient to check for intestinal disorders. Mainz pouch II UD procedure was deemed inappropriate if the result was positive.

The median (range) operative time was 475 (300–750) min, and the postoperative LOS was 13 (7–42) days. Hadzi-Djokic JB et al. [9] and Bastian PJ et al. [22] reported that the mean (range) operation duration was 270 (192–498) min and 300 (225–510) min, and the mean hospital stay was 17.8 (14–45) days and 14.7 (10–31) days, respectively. Our operative time was longer than previously reported, which was in line with that RARC had a longer operative time (428 vs. 361 min, *p* = 0.0005) [23, 24].

Continence is one of the most important functional outcomes for patients undergoing ONB and Mainz pouch II UD. Mainz pouch II UD is associated with excellent continence [7, 9], and our present study reports day- and night-time continence rates of 91.9% and 86.5%, respectively, in keeping with other published series (Table 4). Other published rates of daytime continence vary from 88.2 to 100%, whereas rates of night-time continence range between 82.4% and 100% [20–22, 25–29]. However, the daytime continence rate ranges between 75% and 97%, and night-time continence rates range between 68% and 83% in patients who underwent ONB [30, 31].

**Table 4:** Previous results from mainz II studies

Author	Year	Number of patients	Follow-up, months mean (SD) /median (range)	Continent		Voiding frequency mean (SD) /median(range)	
				By day	At night	Daytime	Night-time
Fisch M et al. [8]	1993	47	20(10)	100%	97.9%	5(2–8)	1(0–3)
Fisch M et al. [26]	1994	72	19 (1–31)	100%	98.6%	5(2–8)	1(0–3)
Fisch M et al. [25]	1996	73	12.7(1–34)	94.5%	98.6%	0–5(64.4%) > 5(35.6%)	0–3(64.4%) 3–6(27.4%) > 6(8.2%)
Obek C et al. [21]	2001	60	31(11–69)	98.3%	98.3%	5.1(1.1)	1.9(0.7)
Nitkunan T et al. [27]	2004	17	76.8(48-103.2)	88.2%	82.4%	-	-
Bastian PJ et al. [22]	2004	41	19(1–80)	100%		1–3(23%) 3–6(42%) > 6(35%)	1–3(61%) 3–6(29%) > 6(7%)
Bastian PJ et al. [20]	2004	31	24.4 (6–84)	100%	100%	0–1 (0%) 1–3 (23%) 3–6 (45%) > 6 (35%)	0–1 (3%) 1–3 (61%) 2–6 (29%) > 6 (7%)
D’Elia G et al. [28]	2004	102	46.2	97%	95%	6(2–9)	2(0–4)
Triantafylidis A et al. [29]	2005	29	23(7–42)	100%	96.6%	-	-
Hadzi-Djokic JB et al. [9]	2006	177	21(1–84)	99%	99%	4.2 (1.6)	2.1 (0.5)
Zheng D et al. [10]	2021	63	15(IQR: 8-27.75)	95.2%	87.3%	-	-
Zheng D et al. [10]	2021	21	15(IQR: 8-27.75)	100%	90.5%	-	-

In our study, the median (range) voiding frequency was 6 (4–10) during the day and 3 (2-5.5) at night, as in other series (Table 4). Most patients void 3–6 times daily and 0–3 times at night [8, 9, 20–22, 25, 26, 28]; the voiding frequency at night is relatively high, but it is not a major trouble in most patients [28]. Hadzi-Djokic JB et al. [9] reported that most patients who received Mainz pouch II UD had a “very good” quality of life.

At present date, the overall mortality rate was 16.7% (6/36), which was consistent with that reported in other published studies. The cause of death was mainly due to tumor progression, and there were no perioperative deaths [5, 9, 21, 27]. The reported mortality rates have been reported at 8.9% (10/112) [27], 12.2% (5/41) [5], 25% (15/60) [21], and 44% (78/177) [9].

We observed early complications in three patients (8.3% (3/36)) and late complications in 7 patients (19.4% (7/36)); Clavien-Dindo grade III-V accounted for 22.9% (8/36) of complications. Obek C et al. [21] reported that early complications occurred in 3.3% (2/60) of patients. Hadzi-Djokic JB et al. [9] reported that early complications occurred in 14% (24/177) of patients and late complications in 8% (14/177) of patients. Bastian PJ et al. [22] reported that the early complication rate was 7% (3/41), and the late complication rate was 16% (5/31). The complication rate of Mainz pouch II UD following RARC appears to be acceptable.

For patients seeking a continent UD for curative intent, Mainz pouch II UD may be a good option for avoiding the need for self-catheterization or an abdominal wall stoma, which are required by ONB or continent cutaneous UD [6]. The ONB is the primary type of continent UD used in practice [3, 6, 7]. However, if a person has urethral invasion, cannot perform self-catheterization, or has recurrent urethral strictures, they may require a Mainz pouch II UD instead [8, 9]. Additionally, the ONB procedure is complex, whereas the Mainz pouch II procedure is relatively simple and quick [9].

Robotic-assisted surgery, a minimally invasive procedure, is being increasingly performed and literature on its use is growing, with 88 articles being published in 2001–2005 and 2018 in 2016–2021 [32], the increase in available literature may be due to developments in urology [32, 33]. Robotics that were initially used for prostatectomy [34] are being increasingly used in urologic and other procedures [34, 35].

The RARC with an intracorporeal approach to Mainz pouch II UD is rarely reported. In the present study, intracorporeal UD was performed successfully; removal of the specimen via the vagina to avoid a large abdominal incision and achieve minimal invasiveness.

During the follow-up period of this study, no neoplasm occurred at the anastomosis of the ureter and bowel. However, every patient should undergo a flexible sigmoidoscopy examination when required or at ten years after surgery and annually thereafter, because the latent period for the development of a neoplasm is at least ten years [36]. The initial neoplasm is seldom malignant; an adenoma develops first and then progresses to adenocarcinoma. It is not known whether the neoplasm originates from the urothelial or intestinal epithelium or from the anastomosis itself.

RC-pentafecta can be used as a standardized composite outcome, and as a potential tool to assess RC quality [18, 37], which includes: ≥16 LNs removed, negative STSMs, absence of major (Grade III-IV) complications at 90 days, absence of long-term UD-related sequelae and clinical recurrence at ≤ 12 months [18]. The survival outcomes of patients who received open RC or RARC were much better than those of their counterparts, achieving RC-pentafecta [17, 18, 37]. According to Laymon M et al. [17], the RC-pentafecta group’s predicted 5-year recurrence-free survival was significantly greater (81.7% vs. 62.5%, *p* < 0.0001). According to Oh JJ et al. [37], the OS rates in the RC-pentafecta group were considerably higher than those in the No RC-pentafecta group (5-year OS 84.4% vs. 76.2%; 10-year OS 70.4% vs. 58.1%; *p* = 0.016). The OS given by Cacciamani GE et al. [18] was observed to be higher in the RC-pentafecta group (*p* < 0.001). Although there was no significant difference (*p* = 0.16), the RC-pentafecta group’s OS in this study tended to be longer (25.6 vs. 21.5 months), maybe as a result of the small number of patients (*n* = 37).

Between 14.7% (50/340) and 53.3% (144/270), the reported RC-pentafecta achievement rate was low [18, 38], while the associated mean (SD) LN count varied between 10.46 (8.6) and 41.3 (19.3) [18, 38]. Missing RC-pentafecta was most often caused by the number of LNs removed < 16 [17]. 32.4% (12/37) of the patients in this study achieved RC-pentafecta, with a mean (SD) number of LNs of 14.84 (9.43). The low number of LNs reduces the RC-pentafecta achievement rate, which could be attributed to the surgeon’s experience. In the study conducted by Cacciamani GE et al. [18] had a mean (SD) of 41.3 (19.3) LNs. The study’s three experienced surgeons performed over 1000 robotic urological cases between them before to the RARC procedure [18]. Nevertheless, our results include data from the learning curve phase.

UD after RC may affect bowel habit [39], the PAC-SYM questionnaire was employed in this study to assess bowel function, albeit it was not a comprehensive tool. Patients who achieved RC-pentafecta had relatively lower bowel function scores, with a mean (SD) PAC-SYM total score, abdominal, rectal, and stool subscores being 8.55 (2.46), 2.73 (1.01), 2.45(1.44), and 3.18 (1.17), respectively (all p > 0.05). PAC-SYM was also utilized in Asanad K et al.‘s prospective study to evaluate bowel function [19]. The results showed that bowel function scores were high three months post-surgery and thereafter declined, indicating that worse symptoms improved over time [19]. However the dynamic changes in scores are outside the scope of this retrospective study. Furthermore, the research’s median scores for PAC-SYM total score, abdominal, rectal, and stool subscores after 23.0 (12.0-36.5) months following surgery were 9.5, 3.1, 2.5, and 3.0, respectively. These scores were higher than those in Asanad K et al.‘s study at 1–3 years and comparable to those at 6–12 months, which were 8.2, 2.8, 1.4, and 9 [19].

The strength of the current study is that using the RC-pentafecta criteria, we first reported the continence status following RARC with Mainz pouch II UD and found that the rate of urinary continence both day and night was relatively higher (91.7% vs. 80.0%, *p* = 0.64) and that those who did had significantly higher satisfaction scores than those who did not (7.3 vs. 5.5, *p* = 0.034).

Meanwhile, the limitations include that the numeric rating system used to assess patient satisfaction was not tested, the study covered just a small number of totally laparoscopic procedures, and the retrospective data collection process was biased and lacked randomization. The results of our use of RARC and intracorporeal Mainz pouch II UD will be reported in the following study.

## Conclusions

In patients with bladder cancer, RARC with Mainz pouch II UD has an excellent continence rate, and attaining RC-pentafecta is related to higher satisfaction scores. Because of its minimal invasiveness, the intracorporeal approach to Mainz pouch II UD is beneficial for female patients.

## Data Availability

The datasets used and/or analysed during the current study are available from the corresponding author on reasonable request.

## Abbreviations

ACCI: Age-adjusted Charlson comorbidity index
ASA: American Society of Anesthesiologists
BCG: Bacillus Calmette-Guérin
BMI: Body mass index
EBL: Estimated blood loss
LOS: Length of hospital stay
LN: Lymph node
MIBC: Muscle-invasive bladder cancer
NMIBC: Nonmuscle-invasive bladder cancer
ONB: Orthotopic neobladder
OS: Overall survival
PAC-SYM: Patient Assessment of Constipation Symptoms
PLND: Pelvic lymph node dissection
RARC: Robotic-assisted radical cystectomy
RC: Radical cystectomy
SD: Standard deviation
TMT: Trimodality therapy
TURBt: Transurethral resection of bladder tumor
UD: Urinary diversion
